# Assessing the fidelity of delivery of an intervention to increase attendance at the English Stop Smoking Services

**DOI:** 10.1186/s13012-016-0498-z

**Published:** 2016-12-28

**Authors:** Molly Sweeney-Magee, Dimitra Kale, Simon Galton, Andrea Hamill, Hazel Gilbert

**Affiliations:** 1 Research Department of Primary Care and Population Health, University College London, Royal Free and University College Medical School, London, NW3 2PF UK; 2 Smokefree Camden (Public Health), NHS Camden, London, UK

**Keywords:** Implementation, Treatment fidelity, Stop smoking clinics, Behaviour change techniques

## Abstract

**Background:**

Implementation fidelity refers to the extent to which a proposed intervention is enacted as designed and is necessary to determine how much the intervention in question is the primary mechanism in any changes observed. Start2quit was a randomised controlled trial that aimed to improve attendance at the English Stop Smoking Service (SSS). The complex intervention combining computer-tailored personal risk letters and no-commitment (“taster”) sessions aimed at encouraging attendance at the SSS doubled attendance at the SSS and significantly increased abstinence rates, although attendance and abstinence varied between participating SSSs. Assessment of the fidelity of the delivery of the taster sessions to the protocol was embedded into the trial and is the focus of this study.

**Methods:**

Eighteen SSSs participated in the study. Taster sessions were delivered by SSS advisors in the area. Of the 131 sessions delivered, 93 (71 %) were recorded and 41 (31.3 %) were selected for transcription and analysis. The taster session protocol contained 73 specified behaviours, which were independently classified into component behaviour change techniques (BCTs) using an established taxonomy for smoking cessation. All transcripts were coded by two authors with 25 % additionally coded by a third. The fidelity of each taster session was expressed as the percentage of overall protocol-specified behaviours that were delivered. Adherence to each BCT was measured as the number of behaviours applied by the advisors within each BCT divided by the total number classified within each.

**Results:**

Adherence of protocol-specified behaviours was relatively high (median 71.23 %), though there was considerable variation (28.76 to 95.89 %) in individual sessions. Median fidelity to specific BCTs across sessions also varied from 50 to 100 %. Shorter sessions, sessions run jointly by two advisors, by female advisors, or by advisors aged 45 to 54 were associated with higher levels of adherence. There was no association between adherence and subsequent attendance at the SSS.

**Conclusions:**

These results suggest that the delivery of the intervention of this study is not likely to have been impacted by issues of fidelity. As such, we can have greater confidence that variability in the main outcome is not due to variability in SSS advisor adherence to the protocol of the taster sessions.

**Trial registration:**

Current Controlled Trials ISRCTN76561916

## Background

Implementation fidelity refers to the extent to which a proposed intervention is enacted as designed [1]. Three core components of implementation fidelity have been proposed: treatment delivery, treatment receipt and treatment enactment [2]. Assessment of each of these elements of fidelity is essential to enable the researcher to assess whether the independent variable has been manipulated as intended [3]. Bellg and colleagues [4] have argued that without knowledge of fidelity, it is impossible to determine how much the intervention in question is the primary mechanism in any changes observed. Recently published guidance from the Medical Research Council has also emphasised the importance of a systemic approach to process evaluation [5].

The assessment of treatment receipt and treatment enactment is a complex process involving the verification of participant understanding of intervention content and the monitoring of participant use of treatment-related strategies in everyday life [2]. Measurement of treatment receipt and enactment can provide important insight into the most efficacious elements of an intervention [6] but it is rarely carried out given the complexity of such assessments. However, the measurement of treatment delivery is a simpler process once there is a clear definition of the intervention and the necessary constituents [7].

Despite this, a systematic review of psychosocial treatments by Perepletchikova and colleagues [8] found that only 3.5 % of studies conducted any evaluation of fidelity of treatment delivery. This failure to account for intervention delivery fidelity may lead to erroneous conclusions that particular interventions are not effective when in reality, they were not implemented as intended, or the acceptance of statistically effective interventions which differ greatly from their initial design [4]. These can have serious financial, scientific and individual health consequences [2]. The severity of these consequences is magnified in evaluations of novel interventions [9] which do not have the benefit of previous research against which results can be compared. If new interventions are found to be successful and adopted in real-world settings, it is essential to determine that findings are due to the full delivery of the study protocol as described in publication [6]. Detailed intervention fidelity assessment is essential for the synthesis of data; those conducting meta-analyses need to know that they are combining studies which are as homogeneous as their research question requires [9].

Recent research has begun to address this issue and to propose methods to monitor the fidelity of delivery within study interventions [4]. In particular, considerable support has been found for the use of objective identification of pre-specified intervention content via audio or video tapes of intervention delivery in the assessment of fidelity [2]. The use of present/absent checklists has been identified as the most reliable method of comparing delivered content to prescribed behaviours [10, 11]. Key to this method of fidelity assessment is the pre-specified intervention content or ‘treatment manual’. A manual containing explicit guidelines about the content and method of delivery of an intervention both increases the likelihood of all providers receiving the same training and information and of the intervention being implemented as designed [4]. The content of this manual can range from the provision of general goals to a script to be followed verbatim by advisors [10]. However, it has been found that the greater the specificity of instructions, the greater the likelihood of high fidelity [9].

In addition to identifying the extent of intervention delivery fidelity, it is also important to understand the ‘essential’ components of an intervention [9, 12]. Behaviour change interventions in particular are often complex with many interacting elements aiming to address different aspects of the behaviour being targeted for change [13]. A taxonomy of behaviour change techniques (BCTs) has been proposed to systematically detail the elements of behaviour change interventions. This has been adapted for use in a wide range of health behaviour contexts from preventing weight gain [14] to encouraging HIV prevention behaviours [15]. In recent years, this taxonomy has also been modified to apply to behavioural support for smoking cessation [16].

In this paper, we report on an assessment of the fidelity to the protocol of one part of a complex intervention of a randomised controlled trial. The Start2quit trial [17] aimed to improve attendance at the English Stop Smoking Service (SSS), a national network of specialist smoking cessation services set up in 1999 by the Department of Health, offering intensive advice and support to smokers wanting to quit, in group or one-to-one sessions. The service is free to smokers. However, the proportion of smokers in England using the SSS has always been low at less than 5 % [18], and the latest figures show a continuing downward trend [19].

Participants of the Start2quit trial randomised to the intervention group received a computer-tailored personal risk letter and an invitation to attend a no-commitment ‘taster session’ to find out more about the support provided by the SSS. These taster sessions were delivered by trained SSS advisors and were based on a detailed treatment manual. Results from the trial showed that this intervention more than doubled the odds of attendance at the SSS during the 6-month period between randomisation and follow-up as measured by SSS records, compared to the control group who received a standard generic invitation to contact the service (17.4 vs 9.0 %, OR 2.12 [1.75–2.57], *p* < 0.001). Seven-day point-prevalent abstinence at the 6-month follow-up, validated by salivary cotinine was also significantly higher in the intervention group (9.0 vs 5.6 %, OR 1.68 [1.32–2.15], *p* < 0.001). The number completing the 6-week SSS course was also significantly higher in the intervention than that in the control group (14.5 vs 7.0 %, OR 2.24 [1.81–2.78], *p* < 0.001). However, some variation was found in both attendance and in 7-day validated abstinence between SSSs. Overall attendance between SSSs varied from 2.1 to 23.1 % and validated 7-day abstinence from 2.1 to 13.4 % [20].

Assessment of the fidelity of the delivery of the taster sessions to the protocol was embedded into the trial and is the focus of this study. The objective was to assess the fidelity of delivery of the sessions and to identify essential elements in terms of BCTs. A total of 73 behaviours were specified in the taster session protocol, and these were classified by the researchers into 17 BCTs (15 from the taxononomy of BCTs and two novel BCTs) [16]. This study aimed to answer the following research questions: (a) to what extent did advisors adhere to protocol-specified content and BCTs, (b) were the characteristics of the advisors or of the sessions related to adherence to protocol-specified content, and (c) was adherence to protocol-specified content related to participants’ attendance at the SSS or to validated 7-day abstinence.

## Methods

### Start2quit study procedure

Eighteen SSSs across England and 99 general practices within the SSS areas were recruited into the trial between February 2011 and October 2013. All current cigarette and roll-up smokers over the age of 16 identified in participating practices (*n* = 106,819) were sent an invitation to participate in the study along with a Smoking Behaviour Questionnaire (SBQ). Smokers returning the questionnaire and giving consent (*n* = 4384) were randomly allocated to the control group (*n* = 1748) or to the intervention group (*n* = 2636). Those in the control group were sent a generic letter advertising the SSS in their area and those in the intervention group were sent a computer-tailored letter signed by their GP containing personalised risk information and an invitation to a no-commitment ‘taster session’ to find out more about the SSS.

### Taster sessions

The goals of the taster session were to offer information about the SSS, to promote the service and to encourage sign up to a course. It was not intended to replicate the first session of an SSS course. A standard protocol of the content of the taster sessions was prepared and a detailed manual produced. Advisors in each SSS, already trained to give smoking cessation advice in group and one-to-one sessions and with previous experience of facilitating SSS programmes, attended a 2-h training session to enable them to facilitate the taster sessions according to the standardised protocol and manual. The training session included a basic introduction to the methodology of randomised controlled trials and of uniformity of an intervention, an explanation and clarification of the study protocol and procedures, and specified the exact information to be delivered in the session. Thus, the importance of standardising taster sessions and of delivering all protocol-specified content was emphasised, while allowing for differences in the organisation of the individual SSSs, and also allowing for advisors to deliver the information naturally, as they would in their smoking cessation clinics.

A total of 146 taster sessions were organised across the 18 SSS areas. Of these, 131 went ahead as planned and 15 were cancelled due to lower than expected recruitment rates. Only trained advisors led the taster sessions, and each session was run either by one advisor with additional administrative support provided by one other, or the presentation was divided between the two advisors, with one advisor leading and the other supplementing some of the content. To assess fidelity to the protocol, the taster sessions were, with the consent of the attendees, audio-recorded.

Of the 131 sessions delivered, 93 were recorded (71 %). The remainder were not recorded due to forgotten recording equipment, equipment failure or no consent for recording by one or more participants attending the session. Due to the quantity and length of the recordings, sessions to be transcribed and analysed were purposively selected to ensure one session by each lead advisor (where available) was included (*n* = 41, 31.3 % of sessions delivered). If there was more than one recorded session available for a lead advisor, one session was selected at random.

To ensure that those selected were representative of the total taster sessions, analysed sessions were compared to those not analysed for session characteristics and outcome variables. There were no significant differences in length of sessions, number of attendees or in outcomes (SSS attendance or 7-day point prevalent abstinence at the 6-month follow-up).

### Attendees

Attendees in this study were defined as those participants of Start2quit who were randomised to receive the intervention and who also attended a taster session included in this analysis (*n* = 222).

### Measures

#### Adherence measures

The taster session protocol contained 73 specified behaviours, all of which were either specific information that the advisors should communicate (e.g. that the first SSS session involves discussion of reasons for and against smoking) or instructions that they should follow (e.g. ask attendees how many of them enjoy smoking). A coding frame was developed by the first author based on this protocol. Two additional researchers verified the frame by checking that it contained all behaviours specified in the taster session protocol.

The behaviours specified in the coding frame were independently classified by two of the authors into component BCTs using an established taxonomy of 45 smoking cessation BCTs [16]. This was conducted by examining the description of each BCT and assessing if any behaviours in the coding frame corresponded with this description. For example, the coding frame behaviours of ‘Making decision in first session after weighing up pros and cons’ and ‘Emphasises that they won’t be told to quit’ were matched to the BCT of ‘Emphasise choice: Emphasise client choice within the bounds of evidence based practice’ (see Table 1 for full list). This was completed by each author separately before coming together to discuss the rational for each behaviour’s classification. Following discussion, it was decided that most of the protocol-specified behaviours were represented by 15 BCTs. The remaining BCTs detailed in the taxonomy by Michie and colleagues [16] were not used as they were not applicable to the taster sessions. Additionally, two novel BCTs were developed to account for the remaining behaviours that did not fit into the existing 45 BCTs proposed by the taxonomy: ‘Promote the SSS’ and ‘Importance of behaviour change’. These BCTs accounted for behaviours that were uniquely important to the aims of the taster sessions, encouraging a commitment to changing behaviour by quitting smoking utilising SSS support (Table 1).

**Table 1:** Protocol-specified behaviours classified into behaviour change techniques

Behaviour change technique^a^	Description	Component behaviours in taster session manual (*n* = 73)
Give information on stop-smoking medication	Explain the benefits of medication, safety, potential side effects, contraindications, how to use them most effectively and how to get them; advise on the most appropriate medication for the smoker and promote effective use	1. Use of medication is an important part of quitting
		2. Nicotine deprivation may lead to withdrawal symptoms
		3. Medication available to reduce cravings while adjusting to not smoking
		4. How NRT works
		5. Types of NRT available
		6. Zyban and Champix and how they can help reduce desire to smoke
Boost motivation and self-efficacy	Give encouragement and bolster confidence in ability to stop	1. Congratulates attendees on coming to the session
		2. Attending session suggests motivation to quit
		3. This an important step in process of quitting
		4. Positives of this, being something to prepare for
		5. Good way of proving that attendees are doing something good for their health
Build general rapport/emphasise empathy of SSS advisors	Establish a positive, friendly and professional relationship with the smoker and foster a sense that the smoker’s experiences are understood	1. Introduces self and describes personal background
		2. Explains understanding of SSS advisors that smoking is something attendees enjoy
		3. Support in event of ‘slip up’
		4. SSS can help work out cause of slip up and work out strategies for avoiding future occurrences
		5. Recap; thank attendees for attending
Elicit and answer questions	Prompt questions from the smoker and answer clearly and accurately	1. Asks for questions
Elicit client views	Prompt the client to give views on smoking, smoking cessation and any aspects of the behavioural support programme	1. Encourages participation
		2. Encourages attendee participation
		3. Encourages participation on withdrawal symptoms
Emphasise choice	Emphasise client choice within the bounds of evidence based practice	1. Making decision in first session after weighing up pros and cons
		2. Emphasises that they will not be told to quit
Explain expectations regarding treatment	Explain to the smoker the treatment programme, what it involves, the active ingredients and what it requires of the smoker	1. SSS supports smokers to stop smoking completely, not to cut down
		2. First session as preparation for stopping smoking
		3. First session involves discussion of reasons for and against smoking
		4. Setting of quit date will be encouraged during first few sessions
		5. Emphasises that weekly contact is extremely important
		6. Explains that this is why weekly contact is so important
Explain purpose of CO monitoring	Explain to the smoker the reasons for measuring CO at different time points, e.g. before and after the quit date	1. Introduces test for CO present in body
		2. Explains its use in SSS courses
		3. Mentions that it will be possible to compare this reading to one they have later at SSS after they quit
Explain the importance of abrupt cessation	Explain why it is better to stop abruptly rather than cut down gradually if at all possible	1. Not a single puff rule and its effectiveness
Give options for support with the SSS	Give information about options for additional support where these are available (e.g. websites, self-help groups, telephone helpline)	1. How many sessions in a course
		2. Courses can be run by SS advisor or practice nurse
		3. Minimum number of sessions following quit date
		4. Gives detail on length of sessions
Identify reasons for wanting and not wanting to stop smoking	Help the smoker to arrive at a clear understanding of his or her feelings about stopping smoking, why it is important to stop and any conflicting motivations	1. Asks attendees how many of them enjoy smoking
		2. Identify reasons for wanting and not wanting to stop smoking
		3. Asks attendees why they are considering quitting smoking
Measure CO	Measure expired-air carbon monoxide concentration	1. Offers attendees opportunity to have CO levels read
		2. Encourages all attendees to have reading taken
Provide info on consequences of smoking and smoking cessation	Give, or make more salient, information about the harm caused by smoking and the benefits of stopping; distinguish between the harms from smoking and nicotine; debunk myths about low tar and own-roll cigarettes and cutting down	1. Short-term benefits of quitting
		2. Long-term benefits of quitting
		3. Explains CO is a poisonous gas contained in cigarette smoke
		4. Explains nature of toxicity of CO
		5. Good news that levels of CO drop very quickly once they stop smoking
		6. Immediately improves circulation and chance of any related health problems
Provide info on withdrawal symptoms	Describe to smokers what are, and are not, nicotine withdrawal symptoms, how common they are, how long they typically last, what causes them and what can be done to alleviate them	1. Asks attendees for any common withdrawal symptoms
		2. Mentions common symptoms if none are suggested by attendees (e.g. stress/anger/lower concentration/increased appetite)
		3. Emphasises that not everyone will experience these symptoms
Summarise information/confirm client decisions	Provide a summary of information exchanged and establish a clear confirmation of decisions made and commitments entered into	1. Recap-Mention that there are benefits to quitting in both long and short term
		2. Recap-Mention that attending a course will make it four times more likely that they will have a successful quit attempt
		3. Recap-Mention the courses will help develop strategies to avoid smoking
		4. Recap-Mention they will also receive information on available medications
		5. Recap-Remind attendees to complete an evaluation form and return it to an advisor
		6. Recap-Emphasise that immediate sign up to a SSS course is possible
Importance of behaviour change^b^	Detail the role habits play in smoking and emphasise the help the SSS can provide in breaking the associations between smoking and situational triggers	1. Explains habitual nature of smoking
		2. Trigger points
		3. Importance of developing strategies to break the association between these trigger points and smoking
		4. SSS support of behaviour change
		5. Emphasises medication not being miracle cure and behaviour change is also needed
Promote SSS^b^	Detail the success rates of the SSS and explain how SSS advisors can help smokers stop smoking and remain quit in the long term	1. Explains SSS is based on well-researched evidence
		2. Attending an SSS course has been proven to be the best way to help people quit
		3. Services are free
		4. Those attending course are four times more likely to stop and stay stopped than those who try and quit on their own
		5. Remaining sessions are for support
		6. Help in developing strategies to avoid smoking is key aspect of SSS course
		7. Able to find out more about NRT at SSS
		8. Advisors can aid in choosing between different forms of NRT
		9. Able to find out more about these medications from SSS
		10. Support available from SSS advisors in this process
		11. Mentions potential sign up
		12. Shows DVD to attendees

^a^From Michie et al. [15]

^b^Novel BCTs not derived from Michie et al. [15]

### Session characteristics

Taster session characteristics included the structure of the session (one advisor or two providing content), the length of the session and number of attendees.

### Advisor characteristics

All advisors were recruited on the basis that they were employed by the SSS and had some previous experience of assisting people to quit smoking. Advisors also completed a short questionnaire at the time of training. Data gathered included gender, age, highest educational qualification, type of smoking cessation training, time since smoking cessation training, employer, job title and number of patients seen in the previous 6 months.

### Attendee outcomes

The proportion of attendees at each session who subsequently attended the SSS, and the proportion of attendees at each session who were found to be 7-day point-prevalent abstinent at the 6-month follow-up, biochemically validated by salivary cotinine, were measured. It is important to note that attendance at the SSS was the behaviour targeted by the taster sessions and the one hypothesised to be related to the fidelity of delivery of the intervention. However, 7-day point-prevalent abstinence data was also included to test the hypothesis that there was no link between this outcome and adherence given the potentially complex pathway between the taster sessions and cessation.

### Procedure and analysis

All transcripts were anonymised for SSS area and advisor. Each transcript was coded by a minimum of two authors; specifically MSM coded all transcripts and DK and AH coded 50 % each. The remaining authors (HG and SG) coded 25 % of the transcripts between them, chosen at random, to provide an extra level of reliability checking. Average inter-rater reliability for coding was 86 % (68–99 %) across sessions. All disagreements were resolved through discussion between researchers. Data from the coding frames were double entered into Excel and discrepancies corrected.

The fidelity of each taster session was expressed as the percentage of overall protocol-specified behaviours that were delivered, that is the number of protocol specified behaviours applied by the advisor divided by the total number of behaviours (for example: number of behaviours applied by the advisor = 50/total number of behaviours = 73 = 68.5 %). Adherence to each BCT was measured as the number of behaviours applied by the advisors within each BCT divided by the total number classified within each.

Mean and median adherence to protocol specified behaviours and for each BCT was calculated. *t* tests and analysis of variance were used to assess differences in adherence to the protocol by advisor characteristics and advising structure. Correlations were computed to explore the association between adherence to protocol-specified behaviours and the length of session. To assess whether adherence was related to the main outcome measures of attendance at the SSS and 7-day point-prevalent abstinence, high and low adherers were split into two groups (high adherers were those sessions that reached the median and above), and *t* tests used to assess differences in outcomes between these groups. Data were analysed in SPSS (v22).

### Ethical approval

The study was approved by the by the South West London Research Ethics Committee, and R & D approval was obtained from all participating SSSs.

## Results

### Session characteristics

Mean session duration was 43 min and 4 s and ranged from 14 min to 1 h and 18 min. Twenty-seven sessions (65.85 %) were facilitated by one lead advisor with minimal administrative support by an additional advisor, and 14 (34.15 %) were split between two advisors with one taking the lead. The number of smokers attending a session ranged from 1 to 17 (mean = 5.41, median/mode = 4).

### Advisor characteristics

Only lead advisor characteristics were assessed due to their leading role in the taster sessions and the significantly greater proportion of the information that they communicated to attendees. The majority of advisors were female (73.17 %) and 51.22 % were aged between 45 and 54. The majority were educated to degree level or higher (73.17 %), 41.46 % had received smoking cessation training to NCSCT (National Centre for Smoking Cessation and Training) stages 1 and 2 with various additional training and 60.98 % had received training within the previous 3 years. All were employed by the SSS and 70.73 % were employed as SSS advisors. Most (90.24 %) had seen 21 or more patients in the last six months (Table 2).

**Table 2:** Characteristics and mean adherence to protocol specified behaviours associated with characteristics of lead advisors in analysed taster sessions (*n* = 41)

	*n*/%	Mean (SD) adherence to protocol specified behaviours	95 % CI of the difference in means	*p*
Gender			−24.85 to −2.49	.018
Female	30/73.17	72.19 (15.83)		
Male	11/26.82	58.53 (15.24)		
Age				.021
18–44	12/29.27	61.87 (17.55)	50.72 to 73.02	
45–54	21/51.22	75.41 (14.3)	68.91 to 81.92	
55+	8/19.51	60.45 (14.96)	47.94 to 72.95	
Highest qualification			−15.76 to 8.17	.525
A level or lower	11/26.83	65.75 (20.17)		
Degree or higher	30/73.17	69.54 (15.43)		
Smoking cessation training^a^				.594
NCSCT stage 1/plus additional training	8/19.51	70.38 (15.82)	57.15 to 83.61	
NCSCT stages 1 and 2 or SCTRP	15/36.59	65.66 (16.24)	56.67 to 74.65	
NCSCT stages 1 and 2 plus additional training	17/41.46	71.55 (17.32)	62.65 to 80.46	
Missing	1			
Time since stage 2 training			−19.98 to 5.51	.257
1–3 years	25/60.98	67.23 (18.71)		
4+ years	11/26.83	74.47 (13.49)		
Missing	5			
Employer				
SSS	100/100	68.63 (16.65)	–	–
Job title				.388
SSS advisor	29/70.73	69.03 (17.78)	62.73 to 75.34	
SSS manager	4/9.76	68.77 (13.4)	52.13 to 85.41	
Healthy lifestyle advisor	2/4.88	93.84 (2.91)	67.73 to 119.94	
Administrator	3/7.32	66.2 (14.27)	30.76 to 101.63	
Other	1/2.44	67.1	–	
Missing	2			
Number of patients in previous 6 months			−22.59 to 18.86	.857
< 21	3/7.32	66.67 (33.68)		
> 20	37/90.24	68.53 (15.62)		
Missing	1			

NCSCT Stage 1 = National Centre for Smoking Cessation and Training Stage 1 certification, NCSCT Stage 2 = National Centre for Smoking Cessation and Training Stage 2 certification

^a^SCTRP = Smoking Cessation Training and Research Programme

### Adherence

**Fig. 1:**
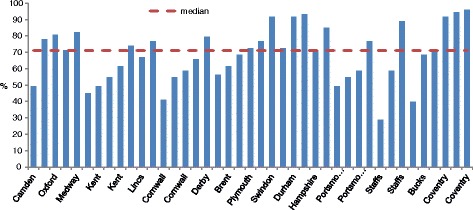
Adherence to manual specified content during each analysed taster session

A median of 71.23 % (*m* = 68.53 %) of protocol-specified behaviours was delivered across sessions, though there was considerable variation. Adherence in individual sessions varied from 28.76 to 95.89 % (Fig. 1). As can be seen in Fig. 1, low adherence sessions were not concentrated in particular SSS areas.

**Fig. 2:**
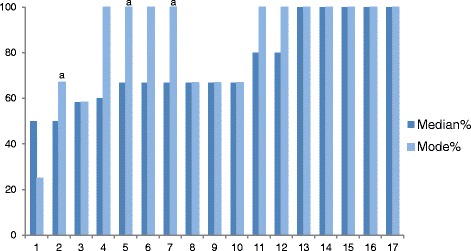
Median and mode fidelity to BCTs across sessions. *a* Multiple modes exist. The highest value is shown. Key to BCTs: *1* give options for SSS support, *2* summarise information, *3* promote the SSS, *4* boost motivation, *5* give information on stop-smoking medications, *6* elicit client views, *7* explain expectations regarding treatment, *8* explain purpose of CO monitoring, *9* identify reasons for wanting/not wanting to quit, *10* provide information on withdrawal symptoms, *11* build general rapport, *12* explain importance of behaviour change, *13* elicit and answer questions, *14* emphasise client choice, *15* explain importance of abrupt cessation, *16* measurement of CO, *17* give information on consequences of smoking

Median fidelity to specific BCTs across sessions also varied from 50 % (‘Summarise information’ and ‘Give options for support’) to 100 % in five of the BCTs (Fig. 2). However, two of the five consisted of only one behaviour, and another two consisted of only two behaviours. The fifth one: ‘Provide information on the consequences of smoking and smoking cessation’ consisted of six behaviours and had the highest adherence. Figure 2 also shows the mode for each BCT and indicates that, with the exception of ‘Summarise information’, sessions included at least some of the specified behaviours included in each BCT.

### Association between adherence and session and advisor characteristics

Sessions where the advisor was assisted by a second advisor were found to have significantly higher adherence levels than sessions run by one advisor alone (75.73 vs 64.79 % [95 % CI for difference −21.61 to −0.29], *p* = 0.044). There was a negative correlation between session length and adherence, thus shorter session length was associated with increased adherence to protocol-specified content (*r* = −.351, *n* = 41, *p* < 0.025).

Female advisors had significantly higher levels of adherence to protocol-specified behaviours than male advisors (72.19 vs 58.53 %, *p* = 0.018). Level of adherence also varied with advisors’ age, those aged between 45 and 54 were significantly more adherent (75.41 %) than advisors who were younger (61.87 %) or older (60.45 %) (*p* = 0.021). Full details including CIs are reported in Table 2.

### Attendee outcomes

The proportion of attendees who subsequently attended the SSS ranged from 0 to 100 % (*m* = 44.94 %), and the mean proportion of attendees who were found to be validated 7-day point-prevalent abstinent at the 6-month follow-up was 20.21 % (range 0 to 66.67 %).

### Association between adherence and attendee outcomes

No differences were found between the high and low in adherence groups in either the proportion of attendees attending the SSS (44 % vs 45.9 %, low vs high groups, respectively) or in the proportion who were 7-day point-prevalent abstinent (22 % vs. 18.6 %, low vs high groups, respectively).

## Discussion and conclusions

This study represents a thorough assessment of intervention delivery fidelity to the taster session protocol of the Start2quit trial. The median adherence to specified behaviours of 71.23 % was high relative to similar evaluations [21, 22]. However, one quarter of sessions fell below 50 %, one as low as 29 %. Overall adherence was greater in sessions which were run jointly by two advisors, in sessions which were run by female advisors and by advisors aged 45 to 54. Additionally, shorter sessions were associated with higher levels of adherence. An assessment of the association between adherence and attendance at the SSS was found to be non-significant.

This study has some important implications for future fidelity assessments of novel interventions. Firstly, the finding that more specific techniques were more likely to be delivered suggests the importance of considering the previous training and expertise of those delivering an intervention. While it has been reported elsewhere that more specific BCTs are less likely to be delivered reliably [23], this was not the case in this study. It is possible that this was due to the experience of participating advisors in delivering smoking cessation courses. This factor may also be related to the relatively high level of adherence found in the analysed sessions; employing individuals who have previous experience with behaviour change strategies may lead to greater intervention delivery fidelity than training the uninitiated in specific study-related BCTs. Additional research is needed to explore this further, particularly in areas targeted for behaviour change other than smoking.

The finding that sessions run by two advisors had higher levels of adherence than those run by a single advisor has potentially significant implications for future implementation assessments. One possible explanation for this result is that the second advisor acted as a safety net, delivering key items that the first advisor failed to present. Research in the field of sociology has suggested that co-facilitation of training sessions can be beneficial due to shared responsibility and mutual support [24, 25]. Thus, presenting in pairs may have mitigated feelings of nervousness for some advisors who had less experience of facilitating groups. Alternatively, having a second facilitator may have created an observant other, or feeling of assessment, increasing attention to the protocol and adherence. Caution should be exercised in relation to the finding that two advisors are more effective, as the content of the second advisor was not analysed. Future research could assess how a second facilitator may improve or aid in fidelity to protocols.

This study also examined specific advisor characteristics. Being female and being aged between 45 and 54 were significantly associated with adherence to the protocol. These findings should be treated with caution due to the small sample size, the fact that not all advisors were included in the analysed sessions, and the omission from the analysis of the contribution of secondary advisors who delivered a minority of the content. However, they do indicate a need for more research into how advisor characteristics potentially influence adherence to study manuals and how this influence can be mediated through training. To date, the exploration of the reasons for variation in adherence has been largely ignored with the exception of some work on the impact of practical issues such as lack of time [26].

While the median adherence to specified behaviours was high, there was considerable variability across study areas suggesting some modifications be made to the training provided to advisors if this intervention were to be adopted. Specifically, it may be beneficial to tailor training to advisor experience levels in delivering behaviour change interventions. In addition, given that the two BCTs with lowest adherence were related to providing information on the SSS itself, greater emphasis on the importance of communicating this practical information may be needed.

However, the lack of association between adherence to BCTs and the outcomes of attending the SSS and 7-day abstinence suggests that absolute fidelity may not be necessary for this intervention to be effective. Absolute fidelity is rarely realistic in real-world settings, and in this intervention, it is important that advisors have some freedom to adapt to the specific needs of the people to whom they are presenting. As discussed by Dusenbury et al. [26], key to deciding the relative importance of fidelity and adaptability may be the complexity and structure of the intervention itself. While this intervention is relatively simple, it is structured, suggesting that a fine balance between fidelity and adaptability is required. If this intervention was to be implemented, continued fidelity assessment and outcome measurement would be important to establish which of the BCTs are fundamental to the intervention, and which can be more extensively adapted in different contexts.

Finally, the lack of association between adherence and study outcomes may have been influenced by the generally high levels of motivation of attendees. Responding to an invitation to a smoking cessation study and then attending a session to find out more about the SSS indicates a high level of interest in both quitting smoking and availing of SSS support to do so. Perhaps this enthusiasm meant that the presentation of protocol-specified content was less important than theorised as attendees were already inclined to sign up for an SSS course. In addition, receiving the first half of the intervention, a personalised risk letter, may have meant that the likelihood of participants changing their behaviour had already increased and as such they were more amenable to attending the SSS or quitting smoking.

A key strength of this study is the robust methodology. All sessions were double-coded and 25 % were triple-coded. Sessions were anonymised for area and advisor to remove potential bias and inter-rater agreement was high for all sessions. The protocols were highly specific and all advisors underwent a 2-h training session. In addition, as previously mentioned, all advisors were experienced in delivering smoking-related behaviour change interventions.

A significant limitation of the study is its evaluation of only a sample of the taster sessions delivered as part of the Start2quit study, due to the prohibitive resources required to transcribe and analyse the additional 47 sessions recorded. However, the 41 sessions analysed represent 31.3 % of the total session number, well over the 20 % minimum recommended by Schlosser [27, 28]. Other limitations include the sole focus on adherence to the protocol and the neglect of more subtle competency related variables. Communication characteristics both content-related (e.g. providing examples to clarify point) and non-specific (e.g. empathetic tone) have been shown to influence the effectiveness of behaviour change interventions [29] and ideally should be evaluated [28]. The frequency and duration of delivery of behaviours was also omitted; this ‘exposure’ measurement may have implications for intervention outcomes [30]. In addition, a number of advisors provided extra information, both relevant and irrelevant. These complex factors were not examined due to the associated high evaluation burden.

We did not assess the other key components of implementation fidelity, intervention receipt and enactment. These additional measurements were outside the scope of this study but could have provided clarity on the efficacy of specific BCTs. Finally, the high rate of unrecorded sessions is an issue and it is possible that when this was due to tape failure, it was not random. For example, less well-organised and prepared advisors may have been less likely to remember to record and operate the machine correctly.

Assessing implementation fidelity is essential in ensuring that research studies are actually measuring that which they claim to be measuring [3, 7]. This is particularly true for novel interventions [9] such as that described in this fidelity assessment. The results of this evaluation indicate that a key aspect of the study intervention is not likely to have been significantly impacted by issues of fidelity. As such, we can have greater confidence that variability in the main outcome of attendance at the SSS is not due to variability in SSS advisor adherence to the protocol of the taster sessions. This emphasises the importance of future research in behaviour change interventions including an assessment of fidelity. These assessments are the only way to draw any firm conclusions about the most effective ways to change behaviour.
